# Presence of Inulin-Type Fructo-Oligosaccharides and Shift from Raffinose Family Oligosaccharide to Fructan Metabolism in Leaves of Boxtree (*Buxus sempervirens*)

**DOI:** 10.3389/fpls.2016.00209

**Published:** 2016-03-01

**Authors:** Wim Van den Ende, Marlies Coopman, Rudy Vergauwen, André Van Laere

**Affiliations:** Laboratory of Molecular Plant Biology, Institute of Botany and Microbiology, KU LeuvenLeuven, Belgium

**Keywords:** inulin, oligosaccharides, stress, RFOs, fructans

## Abstract

Fructans are known to occur in 15% of flowering plants and their accumulation is often associated with stress responses. Typically, particular fructan types occur within particular plant families. The family of the Buxaceae, harboring *Pachysandra terminalis*, an accumulator of graminan- and levan-type fructans, also harbors boxtree (*Buxus sempervirens*), a cold and drought tolerant species. Surprisingly, boxtree leaves do not accumulate the expected graminan- and levan-type fructans, but small inulin fructo-oligosaccharides (FOS: 1-kestotriose and nystose) and raffinose family oligosaccharides (RFOs: raffinose and stachyose) instead. The seasonal variation in concentrations of glucose, fructose, sucrose, FOS and RFOs were followed. Raffinose and stachyose peaked during the winter months, while FOS peaked at a very narrow time-interval in spring, immediately preceded by a prominent sucrose accumulation. Sucrose may function as a reserve carbohydrate in winter and early spring leaves. The switch from RFO to fructan metabolism in spring strongly suggests that fructans and RFOs fulfill distinct roles in boxtree leaves. RFOs may play a key role in the cold acclimation of winter leaves while temporal fructan biosynthesis in spring might increase sink strength to sustain the formation of new shoots.

## Introduction

The first fructan (polyfructosylsucrose) ever discovered was inulin, derived from the rhizomes of *Inula helenium*, a flowering plant (Rose, 1804). Since then, they were not only observed in flowering plants, but were also found to be widespread in bacteria. Their presence is also reported in some fungi, algae, liverworts, and one moss (*Sphagnum* sp). About 45,000 species of angiosperms, approximately 15% of the flowering plants, store fructans (Hendry, 1993). Fructans are water-soluble oligo- and polysaccharides (Wiemken et al., 1986) that are known to occur in the highly evolved orders of the Poales (Poaceae), Liliales (Liliaceae), Asparagales (Alliaceae, Asparagaceae), Asterales (Asteraceae and Campanulaceae), and in the Boraginaceae (Hendry, 1993). Recently, one representative of the Buxaceae, *Pachysandra terminalis* (Japanese Spurge), was also identified as a fructan accumulator (Van den Ende et al., 2011).

Both fructans and raffinose family oligosaccharides (RFOs) can be considered as fructosyl and galactosyl extensions of sucrose (Suc), respectively (Van den Ende, 2013). Dicotyledonous species store linear inulin-type fructans consisting of linear β-2,1 linked fructofuranosyl units while β-2,6 levan-type and mixed-type fructans (e.g., graminans and agavins) predominate in monocots (Van den Ende, 2013). Five different fructan classes can be discerned, and different types of fructosyltransferases (FTs; fructan biosynthetic enzymes) have been characterized that can readily explain their synthesis (Di Bartolomeo et al., 2013). Two different enzymes (1-SST and 1-FFT; Supplementary Figure S1) are required to synthesize the most common and best studied inulin-type fructans as occurring in Asteracean species such as *Cichorium intybus*, *Helianthus tuberosus*, and *Cynara scolymus* (Edelman and Jefford, 1968; Koops and Jonker, 1996; Van den Ende and Van Laere, 1996; Hellwege et al., 2000). During evolution, FTs originated independently (on four different occasions) from different, specific types of vacuolar invertases (Schroeven et al., 2008, 2009; Altenbach et al., 2009; Van den Ende et al., 2011; Van den Ende, 2013). In fructan plants, it is well described that fructan accumulation depends on surpassing a certain species-dependent threshold concentration of Suc (Van den Ende, 2013), triggering a Suc-specific signaling pathway (Ritsema et al., 2009; Van den Ende and El-Esawe, 2014). Small inulin-type fructans, termed fructo-oligosaccharides (FOS) in the food industry, are well-known because of their health-promoting properties including prebiotic and/or immunomodulatory effects (Vogt et al., 2013; Peshev and Van den Ende, 2014; Di Bartolomeo and Van den Ende, 2015).

Raffinose [Raf: α-D-Gal (1,6) α-D-Glc (1,2) β-D-Fru] is considered ubiquitous in higher plants (Keller and Pharr, 1996), which is often, but not always (Vanhaecke et al., 2010), accompanied by stachyose [Sta: α-D-Gal (1,6) α-D-Gal (1,6) α-D-Glc (1,2) β-D-Fru]. The first step in RFO biosynthesis is the production of galactinol (Gol) from *myo*-inositol (Ins) and UDP-galactose (UDPGal), a reaction catalyzed by galactinol synthase (GolS). The biosynthesis of Raf and Sta directly depends on Gol (Supplementary Figure S1). Chain elongation proceeds by the reversible addition of galactosyl units from Gol to Suc and is catalyzed by the consecutive action of raffinose synthase (RafS) and stachyose synthase (StaS) (Lehle and Tanner, 1973; Peterbauer and Richter, 1998; Tarkowski and Van den Ende, 2015).

Besides their function as soluble reserve carbohydrates (Van den Ende, 2013), both fructans and RFOs are believed to counteract stresses, such as cold and drought (Bachmann et al., 1994; De Roover et al., 2000; Taji et al., 2002; Amiard et al., 2003; Hisano et al., 2004; Peters et al., 2007; Tarkowski and Van den Ende, 2015), most probably by stabilizing membranes and/or proteins (Vereyken et al., 2001; Hincha et al., 2002, 2003; Valluru and Van den Ende, 2008). Alternatively, they may also act as scavengers of hydroxyl radicals in particular subcellular compartments (Nishizawa et al., 2008; Van den Ende and Valluru, 2009; Peshev et al., 2013; Matros et al., 2015) and/or act as stress signals (Van den Ende, 2013). This view fits well with the fact that transgenic plants carrying FTs and GolS (accumulating fructans and Raf) become significantly more tolerant to various stresses (Kawakami et al., 2008; Nishizawa et al., 2008; Keunen et al., 2013).

Boxtree (*Buxus sempervirens* L.) is an evergreen shrub or small tree (height: 1–9 m) widely distributed in the Mediterranean region, but the species has also become increasingly popular as an ornamental shrub in Western Europe during the last few decades. Boxtree is well-studied as a medical herb with antibacterial and antiviral properties (Durant et al., 1998). This slowly growing, drought and frost tolerant species (Niinemets and Valladares, 2006; Hormaetxe et al., 2007; Di Domenico et al., 2012) possesses long-lived leaves that can be maintained for at least 5 years, and survives successive winter and summer stresses through its lifespan (Letts et al., 2012). One particular adaptation to stress is its ability to transform chloroplasts into red-brown chromoplasts that can revert to functional chloroplasts in regreening leaves (Koiwa et al., 1986; Hormaetxe et al., 2005). The aim of this work was to investigate water soluble sugar dynamics in boxtree leaves throughout the growing season. We show here for the first time that both inulin-type FOS as well as Raf and Sta accumulate in boxtree leaves at different times in the season, strongly suggesting that both types of soluble oligosaccharides fulfill distinct physiological roles.

## Materials and Methods

### Plant Material and Carbohydrate Analysis

To follow the dynamics of soluble carbohydrates in boxtree leaves, four shrubs (height: 1.75 m; age 9 year, Leuven–Heverlee, Belgium, 50°51′47.0′′ N 4°41′01.2′′ E) were used as the study objects. To minimize accumulative defoliation/wounding effects, four fully exposed small twigs (positioned North, East, South, and West in the canopy; each twig containing 10–12 leaves) were sampled on a fortnightly basis (17 h, sampling at varying heights in the tree) and combined in one sample (i.e., 40–45 leaves). A single older individual (height: 3m; estimated age: 70 year; Langemark-Poelkapelle, Belgium, 50°55′43.83” N, 2°51′15.65”E) with a much larger canopy was also sampled, allowing a longer (up to 13 month) and more frequent (weekly) analysis with minimal effect on the overall integrity of the tree.

To compare mid-summer boxtree leaves from a different climate region, an array of differentially pigmented mid-summer *Buxus* leaves (color varying from green to orange and red-brown) were sampled in Castellane (France, 43° 51′ N; 06° 31′′ E) at July 15 (17 h) of the same year, and leaf carbohydrates were analyzed as described above.

### Carbohydrate Analysis and Identification

One gram of leaf material was homogenized with mortar and pestle under liquid nitrogen. Subsequently 1.5 ml of distilled water was added to this fine powder and mixed. Thereafter, the homogenate was heated at 90°C for 10 min. After cooling to room temperature, the extract was centrifuged at 3000 *g* for 5 min (Eppendorf centrifuge 5415D). A 200 μl sample of the supernatant was passed through a mixed-bed ion-exchange column composed of 0.5 ml bed volume of Dowex-50^®^ H^+^ and a 0.5 ml bed volume of Dowex^®^-1-Acetate. The resins were rinsed six times with 200 μl MilliQ water. The pH of the filtrate was adjusted to pH 6.0 with a small amount of unbuffered Tris (1.0 M). The filtrate was diluted twice and centrifuged at 3000 *g* for 5 min. From this neutral fraction, 25 μl was analyzed with high performance anion-exchange chromatography with integrated pulsed amperometric detection (HPAEC-IPAD) as described (Vergauwen et al., 2000). Conversion factors for glucose (Glc), fructose (Fru), Suc, 1-kestotriose (1 K), 1,1-nystose (Nys), Raf and Sta were obtained by injecting pure compounds from Sigma-Aldrich (St. Louis, MO, US) and TCI Europe nv (Antwerp, Belgium).

Enzymatic hydrolysis of inulin-type FOS with heterologous chicory 1-FEH IIa occurred as described (Van den Ende et al., 2011).

## Results and Discussion

### Boxtree Leaves Accumulate Raffinose, Stachyose, and Small Inulin-Type Fructans

**FIGURE 1:**
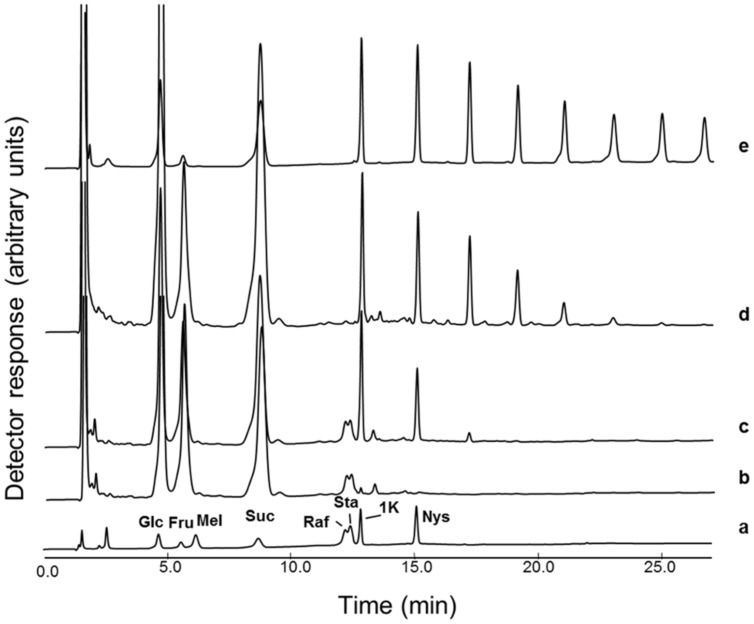
**Buxus leaves can contain both FOS and RFO.** HPAEC-IPAD chromatograms showing **(a)** a reference sample (Glc: glucose; Fru: fructose; Mel: melibiose; Suc: sucrose; Raf: raffinose; Sta: stachyose; 1K, 1-kestotriose; Nys: 1,1-nystose); **(b)** Belgian *Buxus* leaf soluble carbohydrates (see **c**) treated with the heterologous chicory root 1-FEH IIa enzyme (0.1 nkat, 30°C, 2h); **(c)** Belgian *Buxus* leaf soluble carbohydrates (sampled March 26, 60 μg material); **(d)** Mediterranean *Buxus* leaf soluble carbohydrates (July 15, 60 μg material); **(e)** mature chicory root soluble carbohydrates (August 29, 60 μg material).

The carbohydrate pattern of early spring boxtree leaves (sampled in Belgium) showed Raf, Sta, and small inulin-type FOS with a maximal degree of polymerization (DP) of 6 (**Figure 1c**). The nature of the inulin-type FOS and RFOs in Belgian and Meditteranean *Buxus* (**Figure 1d**) was confirmed by comparison with chicory (**Figure 1e**) and sugar standards (**Figure 1a**). Further confirmation was achieved as described before (Van den Ende et al., 2011), by co-elution experiments, mild acid hydrolysis on manually collected peaks and, most convincingly, by incubation of the sample (**Figure 1c**) with heterologous chicory fructan 1-exohydrolase (1-FEH), specifically degrading inulin-type fructans (**Figure 1b**). To the best of our knowledge, RFOs and fructans have never been reported to accumulate together to a high extent within the same plant organ of a particular plant species.

*Buxus sempervirens* shrubs typically accumulate red-colored pigments, both under high and low temperature extremes, and these were proposed to be involved in photoprotection (Hormaetxe et al., 2005, 2007). July is typically a very hot and sunny period in the Mediterranean part of Europe, in contrast to Western-Europe (e.g., Belgium) which has cooler and wetter summers. Interestingly, a preliminary analysis of mid-summer fully irradiated (red-brown colored) boxtree leaves of Mediterranean origin showed more extended Suc and FOS peaks (Supplementary Figure S2C) as compared to half-shaded orange colored leaves (sampled from different plants but in the same area) (Supplementary Figure S2D) while shaded dark-green leaves from the latter plants (Supplementary Figure S2E) and mid-summer leaves of Belgian boxtrees (sampled at exactly the same date in the same year) showed no FOS (see Supplementary Figure S2F). This preliminary experiment, showing a correlation between leaf pigmentation and fructan content, requires further experimental verification. Perhaps the fructans in *Buxus* are also involved in membrane stabilization and hydroxyl radical scavenging processes, as recently proposed for fructans in rapidly growing barley seeds (Peukert et al., 2014).

Intriguingly, Raf and Sta did not accumulate in mid-summer *Buxus* leaves, suggesting a strict cold requirement, as observed in other woody species (Hinesley et al., 1992; Kanayama et al., 2013). Drought stress increased fructan levels, but not the levels of Raf and loliose [α-D-Gal (1,3) α-D-Glc (1,2) β-D-Fru] in *Lolium perenne* parts (Amiard et al., 2003). Clearly, environmental factors can greatly influence carbohydrate content and composition in plants. Therefore, we decided to perform a systematic screening of soluble carbohydrates (Glc, Fru, Suc, RFOs, and small fructans) in boxtree leaves throughout the growing season (see below).

### The Polyphyletic Nature of Fructan Metabolism

The discovery of inulin-type fructans in boxtree was rather surprising for two reasons:

First, despite more than 160 years of fructan research and elaborate screenings among and within plant families (Hendry, 1993), fructan accumulation in boxtree remained undetected. However, it was described before that the soluble carbohydrate fraction was substantially increased in cold-induced leaves (Sieckmann and Boe, 1978), but the exact nature of the saccharides involved was not further investigated.

Second, and contrary to monocots (Valluru, 2015), fructan accumulation in dicot leaves is rather atypical. For instance, Asteracean fructan accumulators such as chicory and *Helianthus tuberosus* only synthesize substantial amounts of fructans when leaves are detached and incubated in exogenous Suc (Van den Ende et al., 2006). However, it was found that leaves of Japanese Spurge, another member of the Buxaceae, accumulate graminan- and levan-type fructans under cold, by the induction of a 6-SST/6-SFT enzyme (Van den Ende et al., 2011; Lammens et al., 2012). Yet, we found no graminan- or levan-type fructans in *Buxus sempervirens*. To the best of our knowledge, the presence of inulin-type fructans has also never been reported in other members of the Buxaceae and in the order of the Buxales.

The Buxales branched off at a very early stage, some 117 million years ago (Anderson et al., 2005), clearly separating them from the Asterales and Dipsacales, two orders in which fructans are widespread (Hendry, 1993). From the results presented here and before (Van den Ende et al., 2011), we show here for the first time that completely different fructan types (inulin versus graminan/levan) can occur in different subclades (*Buxus* and *Pachysandra*) within the same plant family (Buxaceae) (von Balthazar et al., 2000), with absence of inulin-type fructans in *Pachysandra* and absence of graminan/levan type fructans in *Buxus*.

### Seasonal Changes of Soluble Carbohydrates in Boxtree Leaves

**FIGURE 2:**
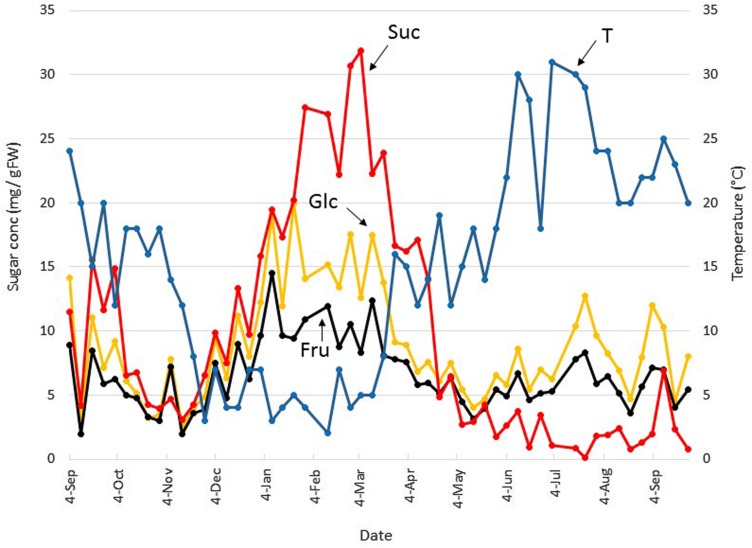
**Seasonal small soluble sugar dynamics in Belgian boxtree leaves.** The concentrations of glucose (Glc, yellow), fructose (Fru, black), and sucrose (Suc, red) in Belgian boxtree leaves from a 70 year old tree (Langemark-Poelkappelle, Belgium) were followed on a weekly basis over a period of almost 13 consecutive months (left *Y*-axis). The mean temperature (T) over the same period is also depicted (blue, right *Y*-axis).

**FIGURE 3:**
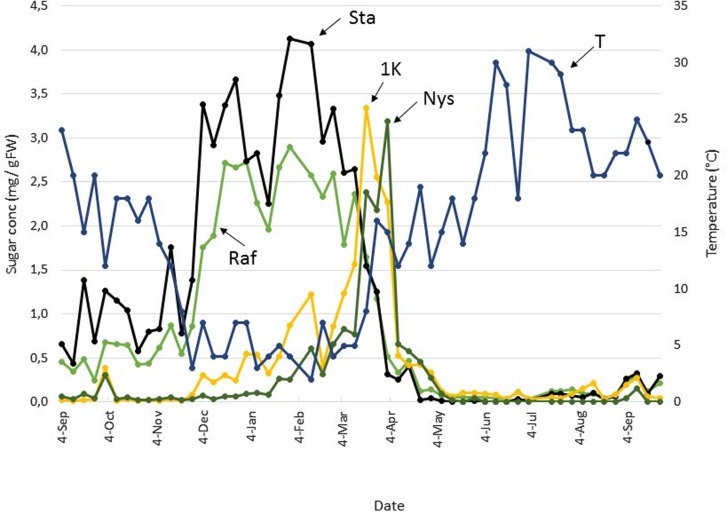
**Seasonal FOS and RFO dynamics in Belgian boxtree leaves.** The concentrations of raffinose (Raf, green), stachyose (Sta, black), 1-kestotriose (1K, yellow), and nystose (Nys, green) in Belgian boxtree leaves from a 70 year old tree (Langemark-Poelkappelle, Belgium) were followed on a weekly basis over a period of almost 13 consecutive months (left *Y*-axis). The mean temperature (T) over the same period is also depicted (blue, right *Y*-axis).

Boxtree leaf soluble carbohydrates were followed on a weekly basis during a time period of more than one year in a large, 70 year old individual (Poelkapelle), starting on Sep 4 (**Figures 2** and **3**). The Glc concentration gradually decreased throughout autumn to reach a minimum (2.2 mg g FW^–1^) on Nov 13 (**Figure 2**). The Glc concentration gradually increased and peaked several times (15 to 20 mg g FW^–1^) in the period Jan-beginning of Mar. After Mar 12, Glc concentrations decreased and remained lower until the beginning of Jul, fluctuating between 5 and 10 mg g FW^–1^. Afterward, Glc concentrations peaked again at Jul 23 and Sep 3, but the concentrations remained lower as compared to the winter months. Overall, the pattern of Fru was very similar to that of Glc (**Figure 2**), and no drastic changes were observed in the Glc/Fru ratio. However, a Glc/Fru ratio peak was detected at Jan 22 (not shown), just before a major increase in Suc concentration, suggesting that Fru was very efficiently phosphorylated (to fructose 6-phosphate) to fuel Suc biosynthesis. Both the pattern and the absolute concentrations of Suc resembled those of the hexoses over the period Sep 4 to Jan 22 (**Figure 2**). From this point in time, hexose concentrations remained roughly constant but Suc concentrations increased gradually to reach an absolute maximum of 32.0 mg g FW^–1^ at Mar 5. During early spring, Suc decreased gradually but steeply, while the temperatures increased steeply. At Apr 23, the concentration of Suc became similar to the hexose concentrations. During the summer period, Suc concentrations further decreased, even becoming lower than the hexose concentrations.

Raf and Sta concentrations fluctuated but remained rather low throughout the period Sep 4 to Nov 20 (**Figure 3**). During this period, the 1K (1-kestose or 1-kestotriose, DP3 inulin-type fructan) and Nys (1,1-nystose or 1,1-kestotetraose, DP4 inulin-type fructan) concentrations remained very low and roughly constant, with the exception of a very small increase at Oct 2. After Nov 20, temperatures decreased, and Raf and Sta concentrations increased alongside, to reach a first maximum at Dec 25. 1K and Nys concentrations also slightly increased during this period but their concentration remained lower compared to Raf and Sta. After a warmer period with temperatures >5°C at the beginning of Jan, Raf, and Sta decreased temporarily but this was not accompanied by decreases in 1K and Nys. A second optimum of Raf and Sta accumulation correlated with a next colder period. Maximal RFO concentrations (2.9 and 4.1 mg g FW^–1^ for Raf and Sta, respectively) were recorded at the end of Jan. The 1K and Nys concentrations increased steadily during Jan and Feb. Starting from Feb 12, Raf and Sta gradually decreased throughout spring. By Apr 2, the concentrations were similar to those observed in the period Sep-early Nov. Interestingly, the sharp decrease in Raf and Sta between Mar 12 and Mar 19 was accompanied by a sharp increase in 1K and Nys. It remains to be noted that this period was preceded by a Suc maximum at Mar 5 (**Figure 2**) and followed by the generation of new shoots which rapidly appeared by the end of Mar/early Apr. At the beginning of Apr, Raf, Sta, 1K and Nys concentrations decreased altogether. A tendency was observed for a change in the 1K/Nys and Sta/Raf ratios (switching from >1 to <1). During the summer period, the concentrations of RFOs and fructans concentrations became very low; with Sta and Nys even becoming undetectable in some samples.

In conclusion, the most drastic changes in RFO and fructan dynamics were observed within the early Dec-Apr period. This time window was subsequently sampled on a fortnightly basis in 4 younger individuals within the same area (Leuven–Heverlee). Overall, somewhat lower absolute levels but otherwise similar Suc, Raf, Sta, 1K, and Nys patterns were obtained (**Table 1**), suggesting that these sugar dynamics do not depend on tree age, but correlate with colder periods (RFOs) and fast spring regrowth (fructans), respectively.

**Table 1:** Evolution of the levels (mg g FW^–1^) of Suc, RFOs (Raf and Sta) and small inulin-type fructans (1K and Nys) in Leuven boxtree leaves during winter and spring.

	Dec 12	Dec 26	Jan 9	Jan 23	Feb 13	Feb 27	Mar 13	Mar 27	Apr 10
Suc	4.9 ± 0.5	8.4 ± 1.0	11.2 ± 2.0	15.4 ± 2.8	18.9 ± 2.1	20.3 ± 3.0	16.8 ± 2.0	11.9 ± 2.9	9.1 ± 1.2
Raf	1.2 ± 0.1	1.6 ± 0.2	1.8 ± 0.3	2.0 ± 0.4	1.8 ± 0.3	1.8 ± 0.3	1.4 ± 0.1	0.7 ± 0.1	0.24 ± 0.04
Sta	1.9 ± 0.2	2.19 ± 0.24	2.3 ± 0.4	2.5 ± 0.4	2.9 ± 0.3	2.5 ± 0.5	1.6 ± 0.2	0.8 ± 0.2	0.18 ± 0.02
1K	0.16 ± 0.01	0.17 ± 0.02	0.38 ± 0.06	0.36 ± 0.06	0.78 ± 0.09	0.6 ± 0.08	1.1 ± 0.1	2.2 ± 0.4	0.37 ± 0.05
Nys	0.028 ± 0.003	0.042 ± 0.005	0.07 ± 0.01	0.18 ± 0.03	0.35 ± 0.04	0.47 ± 0.07	0.63 ± 0.07	1.8 ± 0.4	0.53 ± 0.07

*Standard errors of the mean are presented (n = 4).*

### A Role for Total Water Soluble Carbohydrates and RFOs in Stressed Winter Leaves?

Overall, not much research has been devoted on sugar metabolism in evergreen leaves during the winter period. However, it is well-known that the photosynthetic apparatus is adapted for the survival of frost in a down-regulated state, with potential for photosynthetic activity in winter during periods of permissive temperatures and availability of liquid water (Hormaetxe et al., 2007; Wyka and Oleksyn, 2014).

Likely, a particular slow grower such as boxtree (Letts et al., 2012), will cease all growth activities at temperatures below 5°C. Such low temperature growth arrest may be even universal among all higher plants (Körner, 2003, 2015), where photosynthesis at 5°C is usually operating at 50–70% of its maximal capacity (Körner, 2015), although it is lower in *Buxus* where effective photoinhibition occurs (Hormaetxe et al., 2007). Moreover, woody evergreen species such as boxtree lack typical storage (sink) organs as occurring, e.g., in sugar beet (suc accumulator), chicory (fructan accumulator), and potato (starch accumulator) and therefore it seems favorable to store excess sugars locally in leaves. Therefore, it is reasonable to assume that evergreen leaves could function as a reserve organ, steadily accumulating photosynthetically derived Suc and RFOs toward the end of the winter and early spring period (**Figure 2**) and as such help the plants to acclimate under cold.

It is now recognized that all soluble sugars, also including fructans and RFOs, act as hydroxyl radical scavengers in plants (Nishizawa et al., 2008; Peshev et al., 2013; Peukert et al., 2014; Matros et al., 2015 and references therein). The recent finding that chloroplasts can import Raf from the cytoplasm (Schneider and Keller, 2009) implicates a putative advantage of RFOs as compared to fructans, since no fructan transporters have been discovered so far in higher plants. It can be speculated that a mixture of Suc, Raf, and Sta, together with the protective action of pigments (Hormaetxe et al., 2005, 2007), may contribute to the stability and integrity of chloroplasts during winter, and this presents an interesting area for future research. Note that the total concentration of soluble carbohydrate in boxtree leaves reached a maximum of 66.5 mg g FW^–1^ on Feb 26. For comparison, this is much higher than the one obtained for summer-derived chicory leaves (5.5 mg g FW^–1^; De Roover et al., 2000). Some other tissues specialized in storing soluble reserve carbohydrates are chicory roots and winter leaves of *Ajuga reptans* (Bachmann et al., 1994; Van den Ende and Van Laere, 1996), both accumulating carbohydrate concentrations up to 200 mg g FW^–1^. Sugar beet hypocotyls contain Suc concentrations up to 125 mg g FW^–1^ (Rosenkranz et al., 2001).

Although cold acclimation responses may not be fully comparable between fast-growing herbaceous species and trees, it is interesting that the concentrations of Raf and Sta in cold-acclimated boxtree leaves are 5-8 times higher than the Raf concentration in cold acclimated *Arabidopsis* leaves (0.5 mg g^–1^ FW; Taji et al., 2002) but lower than the Raf and Sta concentrations in cold acclimated *Ajuga* leaves (about 7.5 and 35 mg g^–1^ FW; Bachmann et al., 1994). Raf and Sta concentrations were roughly inversely correlated with the temperature profile (**Figure 3**) which is in accordance with other reports showing that RFOs accumulate during cold acclimation (Taji et al., 2002; Tapernoux-Luthi et al., 2004; Thomas et al., 2004; Peters and Keller, 2009; Tarkowski and Van den Ende, 2015 and references therein).

In *Arabidopsis*, it was recently demonstrated that a high capacity for Suc synthesis is an important parameter during the early stages of cold acclimation (Nägele et al., 2012). Overall, a highly regulated interplay of enzymatic reactions and intracellular transport processes appears to be a prerequisite for maintaining carbohydrate homeostasis during cold exposure and allowing cold acclimation in *Arabidopsis* (Nägele and Heyer, 2013).

### Sucrose: A Central Player in Buxus Leaves?

Both fructan and RFO metabolism need Suc as a substrate (Tarkowski and Van den Ende, 2015). Next to its well-known role as reserve carbohydrate (sugar beet, sugar cane), and major transport sugar in plants (Ruan, 2014), Suc is also believed to act as a stress protectant in higher plants (Guy et al., 1980; Hoekstra et al., 1989; Keunen et al., 2013).

In *Buxus* leaves, Suc makes up 46% of the total carbohydrate in winter (Jan-Mar). Thus, besides Raf and Sta, Suc represents the most prominent carbohydrate in *Buxus* leaves during the winter period (**Figure 2**; **Table 1**). During this period, part of the Suc pool is probably used to produce Raf and Sta, while the fructan concentrations remain surprisingly low despite the strongly increased Suc levels. In severe contrast, both cold and high Suc concentrations have been demonstrated to greatly stimulate fructan metabolism in temperate grasses (Hisano et al., 2004; Bhowmik et al., 2006). This indicates that fructan anabolism in *Buxus* – despite the presence of Suc as a typical signal to trigger FT gene expression (Van den Ende and El-Esawe, 2014 and references therein) – is suppressed in winter, in favor of RFO metabolism, which contrasts to the situation in monocots (Yoshida et al., 1998). In conclusion, while Suc is the most prominent soluble sugar serving as a substrate for RFO synthesis, it seems to not be able to function as a signal to trigger fructan synthesis in *Buxus* during winter.

### Specific Roles for Fructans During Boxtree Sprouting in Spring?

In Belgian boxtree, fructans only peaked during a very narrow time-period in spring, when RFO concentrations steeply decreased (**Figure 3**; **Table 1**), at a time point when Suc concentrations were already decreasing (**Figure 2**; **Table 1**). The switch from RFO to fructan metabolism during spring points at a highly coordinated control of both metabolisms in *Buxus*. It could be speculated that Gol and/or RFOs might act as signals to suppress fructan anabolism under cold, an intriguing future research area.

The remarkable shift from RFO to fructan metabolism in early spring (**Figure 3**; **Table 1**), immediately preceding the onset of bud swelling and new shoot formation in early Apr, suggests that fructan synthesis in new sinks helps to maintain a suitable Suc gradient between source and sink tissues. The intriguing question remains why *Buxus* uses RFOs in winter and fructans in spring, and not the other way around. Fructan and Raf metabolisms differ (Supplementary Figure S1; Tarkowski and Van den Ende, 2015). For the production of one mole of Sta, net one mole of Suc and two moles of UDPGal are required. The synthesis of 2 UDPGal from Suc costs 4 ATP equivalents (2 as UTP), which means that 2 Suc and 4 ATP are consumed to create Sta. On the contrary, 3 moles of Suc are consumed to synthesize 1 mole of Nys (Supplementary Figure S1), and 3 ATP equivalents are required during sucrose re-synthesis (Tarkowski and Van den Ende, 2015). Thus, it can be speculated that fructan synthesis may be an energetically favorable option as compared to RFO synthesis, at times when ATP maximization and optimal use of ATP is essential to support rapid spring regrowth, a situation which clearly differs from the winter status, associated with growth arrest and (partial) photoinhibition. Despite photoinhibition, leaf sugar accumulation occurs, indicating that there is the potential for ATP production to sustain the more costly RFO synthesis during winter.

## Conclusion

Small inulin-type fructans were discovered in leaves of *Buxus sempervirens*, a stress tolerant member of the Buxaceae belonging to the basal eudicots. Surprisingly, this fructan type differed from the graminan- and levan-type fructans detected in *Pachysandra terminalis* within the same family, demonstrating that the polyphyletic nature of fructan metabolism even extends down to the family level. Intriguingly, both inulin-type fructans and RFOs occurred in *Buxus* leaves, but they peaked at different timings of the year. RFOs peaked in winter leaves, and a peculiar switch from RFO to fructan metabolism was observed in early spring leaves, associated with new shoot formation, suggesting that fructan synthesis may link to the creation of sink strength, with fructans potentially being involved in membrane protection and cellular ROS homeostasis, as recently suggested in developing barley kernels (Peukert et al., 2014).

## Author Contributions

WVdE, AVL, and MC designed the experiments. MC and WVdE sampled the material. MC, WVdE, and RV analyzed the carbohydrates. WVdE wrote the manuscript with input from the other authors.

## Conflict of Interest Statement

The authors declare that the research was conducted in the absence of any commercial or financial relationships that could be construed as a potential conflict of interest.
