# Supplementary material for: Presence of Inulin-Type Fructo-Oligosaccharides and Shift from Raffinose Family Oligosaccharide to Fructan Metabolism in Leaves of Boxtree (Buxus sempervirens)
Source: Front Plant Sci. 2016 Mar 1;7:209. doi: 10.3389/fpls.2016.00209 (PMC4771763; doi:10.3389/fpls.2016.00209)
Supplement: Supplementary file 1 [file Image_1.PDF]

## Supplemental figures

### Figure S1

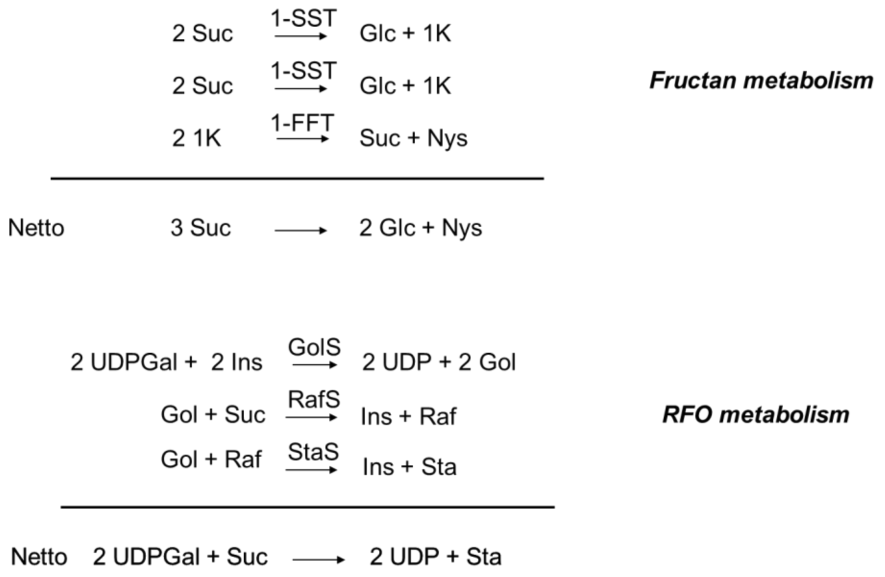


**Fig. S1 Comparison of RFO and fructan synthesis in dicot plants**

1-SST and 1-FFT are typically involved in fructan anabolism in dicot species. 1-SST is the initiator enzyme producing 1-kestotriose (1K) from 2 Suc molecules. Two 1K molecules are required to produce Nystose (Nys), also termed 1,1-kestotetraose. Netto, 3 moles of Suc are consumed to produce 1 mole of Nys. GolS, RafS and StaS activities control plant RFO anabolism. GolS produces 2 molecules of galactinol (Gol) that are used as donor substrate by RafS and StaS, to produce raffinose (Raf) and stachyose (Sta). Netto, 1 mole of sucrose (Suc) is consumed to produce 1 mole of the DP4 oligosaccharide Sta. GolS: galactinol synthase; RafS: raffinose synthase; StaS: stachyose synthase; 1-SST: sucrose: sucrose 1-fructosyltransferase; 1-FFT: fructan: fructan 1-fructosyltransferase; Ins: inositol.

**Figure S2**

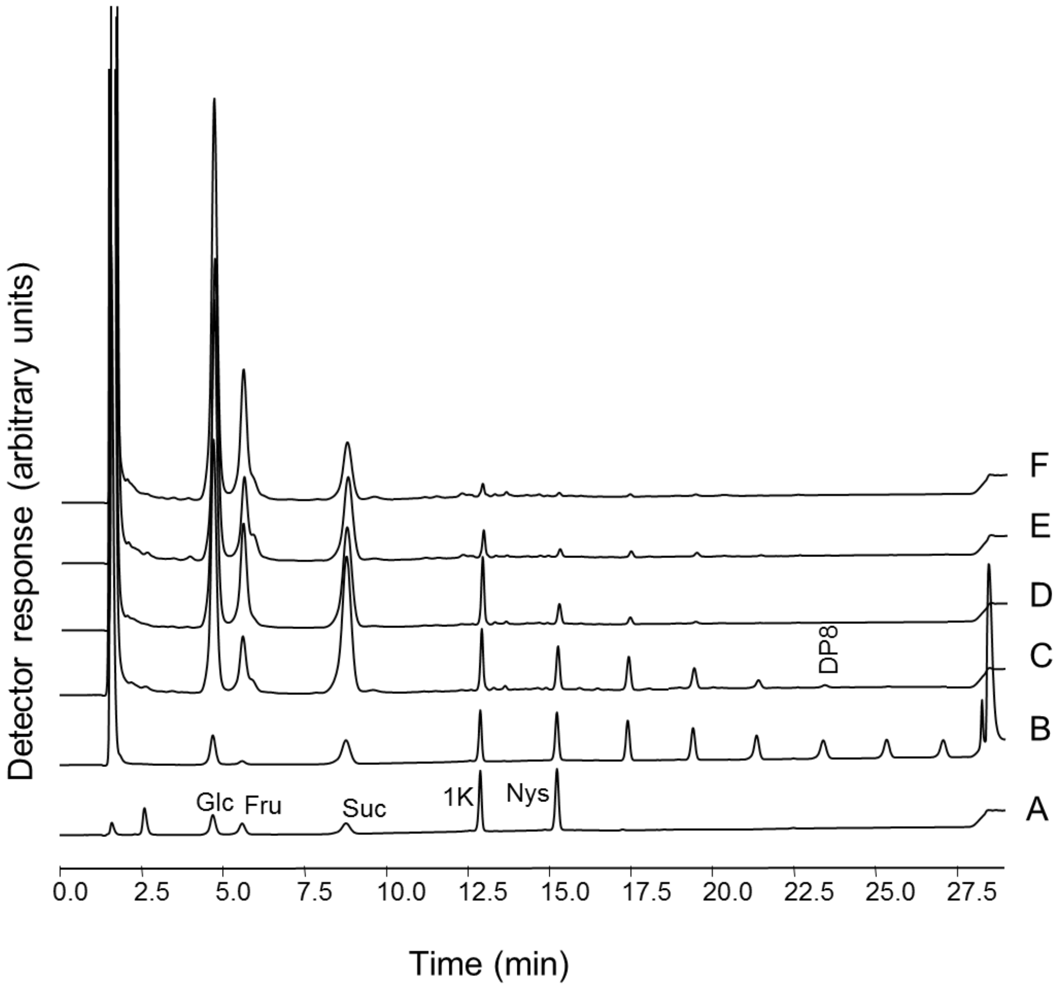


**Fig. S2 Buxus leaf pigmentation and presence of FOS**

HPAEC-IPAD chromatograms with soluble carbohydrates derived from mid-summer Mediterranean *Buxus* leaves with different leaf pigmentation harvested Jul 15. (A) Reference sample (Glc, glucose; Fru, fructose; Suc, sucrose; 1K, 1-kestotriose; Nys, 1,1-nystose); (B) Mature chicory root soluble carbohydrates; (C) Brown-red leaves originating from fully exposed (heat, high light) Mediterranean shrubs; (D) Orange leaves from partly exposed Mediterranean shrubs; (E) Green leaves from partly exposed Mediterranean shrubs; (F) green leaves from shaded Belgian shrubs. DP8: degree of polymerization 8.
